# Combination of acupuncture and Chinese herbal formula for elderly adults with mild cognitive impairment: protocol for a randomized controlled trial

**DOI:** 10.1186/s13063-019-3212-z

**Published:** 2019-02-11

**Authors:** Yueqi Chen, Wenjing Zhang, Huangan Wu, Lixing Lao, Jian Xu, Shifen Xu

**Affiliations:** 1 Shanghai Municipal Hospital of Traditional Chinese Medicine, Shanghai University of Traditional Chinese Medicine, Shanghai, 200071 China; 2 Shanghai Research Institute of Acupuncture and Meridian, Shanghai University of Traditional Chinese Medicine, Shanghai, 200030 China; 3 School of Chinese Medicine, The University of Hong Kong, Pok Fu Lam, Hong Kong; 4 University of Maryland School of Medicine, Baltimore, MD 21201 USA

**Keywords:** Mild cognitive impairment (MCI), Acupuncture, Chinese herbal formula, Protocol, Randomized controlled trial (RCT)

## Abstract

**Background:**

Mild cognitive impairment (MCI) is known as a transitional status between normal cognitive function and Alzheimer’s disease (AD). Acupuncture and Chinese herbal medicines (CHMs) are considered to be beneficial to patients with cognitive impairment. However, it is still unknown whether the combination of the two therapies could optimize the therapeutic effect for MCI. This trial is aimed to evaluate the therapeutic effects of acupuncture and the herbal formula *Yishen* Granule (YSG) for elderly patients with MCI.

**Methods/design:**

This is a multi-sited, patient-blinded, randomized controlled trial (RCT). Two hundred and forty eligible patients will be randomly divided into four groups: A. acupuncture with YSG, B. acupuncture with placebo herbal medicine, C. sham acupuncture with YSG or D. sham acupuncture with placebo herbal medicine. Acupuncture treatment will be given twice a week for 8 weeks and then once a week for 4 weeks. The herbal treatment patients will be given granules daily for 12 weeks, 8 weeks of standard-dose followed by 4 weeks of mid-dose. The primary outcome is scored by the Montreal Cognitive Assessment (MoCA). The secondary outcomes will be scored by the Mini-Mental State Examination (MMSE) and event-related potential (ERP). All the assessments will be conducted at baseline, and at the eighth and 12th week after intervention starts. The follow-up assessments will be performed with the MoCA in the 12th, 24th, and 36th weeks after intervention ends. Intention-to-treat (ITT) analysis will be used in this RCT.

**Discussion:**

This RCT will provide us information on the effect of treating MCI patients with only acupuncture, herbal formula as well as the combination of both. The additive effect or synergistic effect of acupuncture and Chinese herbal formula will then be analyzed.

**Trial registration:**

This trial is registered with ChiCTR-INR-17011569 on 5 June 2017, and has been approved by the Ethics Committee of Shanghai Municipal Hospital of Traditional Chinese Medicine (2017SHL-KY-05).

**Electronic supplementary material:**

The online version of this article (10.1186/s13063-019-3212-z) contains supplementary material, which is available to authorized users.

## Background

Dementia is inevitably associated with an aging population. The progressive deterioration of cognitive function increasingly interferes with daily activity in the elderly [1]. Worldwide, nearly 25 million people have been diagnosed with dementia, which is a major public health problem [2]. In recent years, with the increasing trend of an aging population, the prevalence of dementia has increased year by year. Alzheimer’s disease (AD) is one of the major sub-types of dementia among the population aged 60 years and older in China, and the prevalence of AD increased significantly from 1980 to 2004 [3].

Mild cognitive impairment (MCI) is known as a transitional status between normal cognitive function and clinically probable AD [4]. People with MCI constitute a high-risk group for AD and showed increasing prevalence among older age groups [5]. Subjective cognitive complaints has been regarded as one of the most common presenting symptoms of cognitive impairment, and a prevalence rate of 31.40% for MCI was found in adults aged 50 years and older with subjective cognitive complaints [6]. Effective interventions may attenuate the MCI and the potential risk of a deterioration of the MCI [7]. At present, no pharmacological treatment has demonstrated convincing effects in delaying longer-term progression or conversion to dementia [8].

Acupuncture, a Traditional Chinese Medicine treatment, has been widely used in clinical practice for several thousand years, including nervous system disorders. Basic studies have reported that acupuncture may have effects on multi-infarct dementia and AD via improving memory ability [9, 10]. Several clinical trials have shown that acupuncture at specific acupoints such as *Taichong* (LR3), *Hegu* (LI4), and *Taixi* (KI3) can activate certain cognitive-related regions of the brain in patients with MCI [11, 12]. Moreover, different insertion depths and acupoints may exert different effects [13, 14]. A systematic meta-analysis involving 568 subjects pooled from five randomized controlled trials (RCTs) of acupuncture versus nimodipine treatment has shown that acupuncture is beneficial for amnestic mild cognitive impairment (AMCI) and the combination therapy appears to have superior efficacy [15].

Chinese herbal medicine (CHM) has a long history for treating memory disorders too. Many CHMs, such as *Herba cistanches* and *Polygonum multiflorum*, have shown positive therapeutic effects on cognitive impairment [16, 17]. CHMs are usually used in combination. A placebo-controlled randomized trial showed that the Compound Chinese Medicine, *Bushen* capsule, could improve or maintain the general cognitive function of patients with AMCI during a 2-year treatment [18].

Both the acupuncture and CHMs are considered to be useful in the treatment of MCI. However, it is still unknown whether the combination of the two therapies could optimize the therapeutic effect. The aim for this RCT is to clarify the therapeutic effect of acupuncture combined with CHM for MCI.

## Methods/design

### Setting and design

**Fig. 1:**
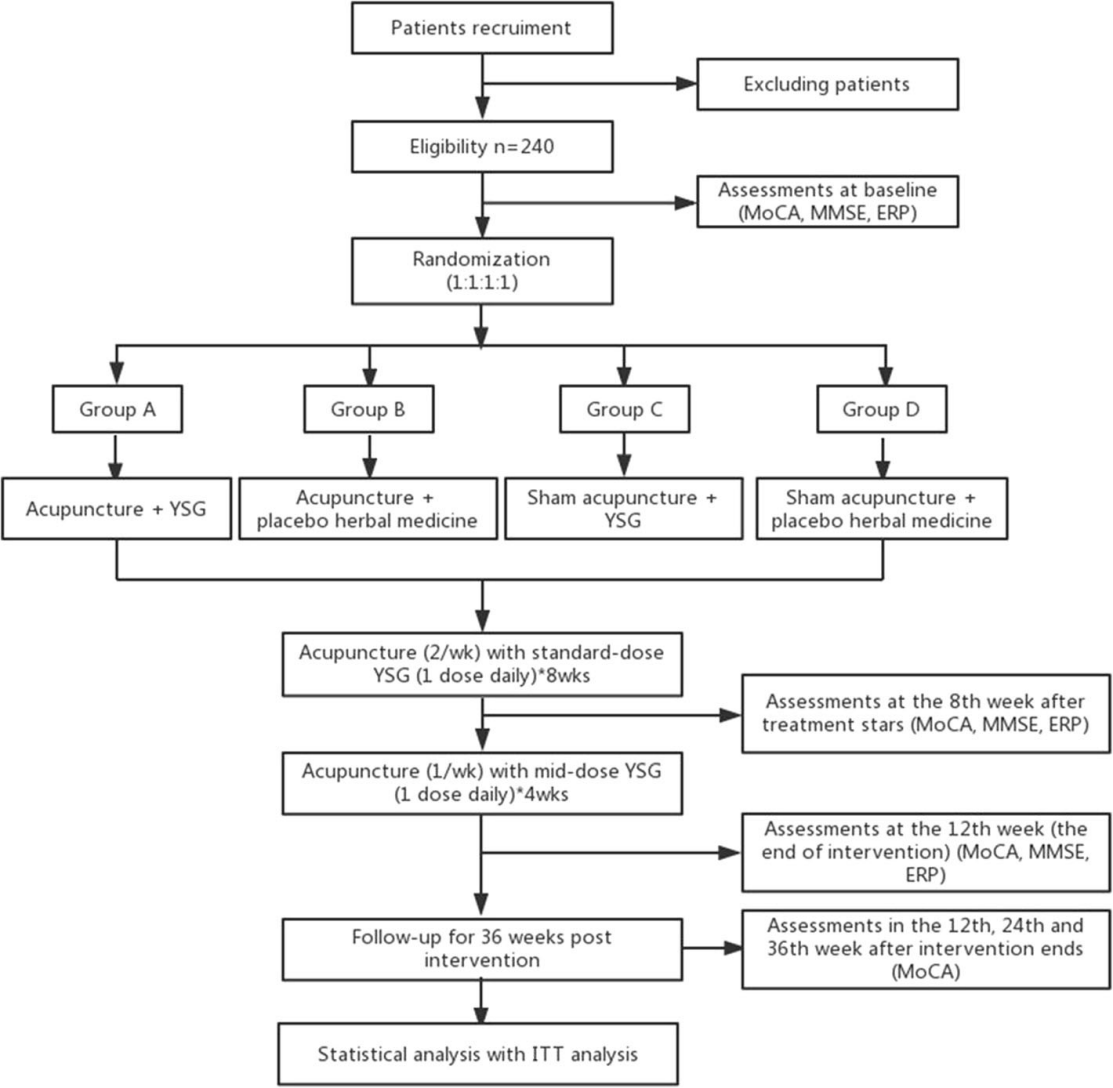
Flowchart of the trial. Figure shows the flowchart of the study process

**Fig. 2:**
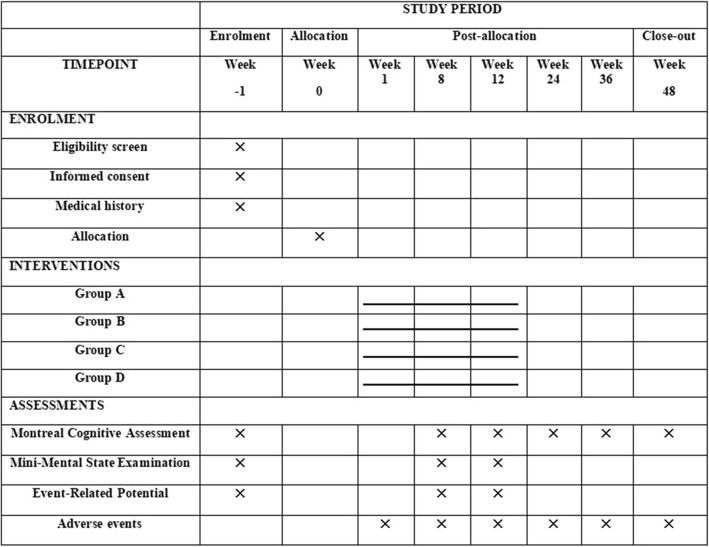
Standard Protocol Items: Recommendations for Interventional Trials Statement (SPIRIT) Figure. Figure shows the enrollment, interventions, and data collection

A multi-sited, patient-assessor-blinded, RCT is designed to evaluate the efficacy and safety of acupuncture and the herbal formula, *Yishen* Granule (YSG), for elderly patients with mild cognitive impairment. Eligible patients will be randomly divided into four groups in a 1:1:1:1 allocation ratio, receiving acupuncture with YSG, acupuncture with placebo herbal medicine, sham acupuncture with YSG, or sham acupuncture with placebo herbal medicine. All the participants will be recruited in Shanghai Municipal Hospital of Traditional Chinese Medicine, Shanghai East Hospital, or Jingan District Hospital of Traditional Chinese Medicine. Written informed consent will be obtained from all patients. The primary outcome is scored by the Montreal Cognitive Assessment (MoCA). The secondary outcomes will be scored by the Mini-Mental State Examination (MMSE) and event-related potential (ERP). All the assessments will be conducted at baseline, and at the eighth and 12th weeks after the intervention starts. The follow-up assessments will be performed with MoCA in the 12th, 24th, and 36th weeks after intervention ends. The flowchart of the study process can be seen in Fig. 1, and the timing of treatment visits and data collection can be seen in Fig. 2 and Additional file 1.

#### Inclusion criteria

Patients meeting all of the following inclusion criteria will be included:

1. Aged 65–85 years; male or female
2. Complaint of allomnesia, which can be obtained from an informant who knows the patient well
3. Essentially intact activities of daily living
4. Global Deterioration Scale (GDS) score 2–3, Clinical Dementia Rating (CDR) score = 0.5, MMSE score ≥ 24, Dementia Rating Scale (DRS) score ≥ 123
5. Able to understand and complete the scale evaluations
6. Agree with the investigation and sign the written informed consent form

#### Exclusion criteria

Participants meeting any one of the following exclusion criteria will be excluded:

1. Participants who are assessed as normal or dementia by the CDR
2. Participants with a HAMD (Hamilton Depression Scale) score > 8
3. Participants with schizophrenia, schizoaffective disorder, primary affective disorder (including the history of affective disorder), or participants with a severe neural disorder (such as aphasia or agnosia)
4. Participants who were diagnosed with AD, central nervous system infections, post-traumatic dementia, toxic encephalopathy, metabolic encephalopathy, Huntington’s disease, multiple sclerosis or Parkinson’s disease before memory decline, or participants with untreated primary endocrine disease
5. Participants with severe primary disease of the cardiovascular, hepatic, renal, or hematopoietic systems
6. Participants suffering from gastrointestinal diseases that affect drug absorption (such as severe dyspepsia, gastrointestinal obstruction, or gastric ulcer)
7. Participants who are allergic to the study granule
8. Participants who have taken Western and/or Oriental medicine in the past 4 weeks to improve their cognitive function

### Sample size

The calculation of the sample size in this trial was based on the change of the MoCA score. Referring to the related trial published in *BMC Complement Alternative Medicine* [7], 80% of patients with MCI showed an improvement of at least 12.5% on the MoCA score after acupuncture treatment. We assumed the rate to be 80% with one active and one control intervention, 85% with both active interventions and 60% with both control interventions. Under the assumption of a significant level of 0.05, 80% power, and 20% dropout rate, a total of 240 participants should be recruited for this RCT (60 participants in each group with 1:1:1:1 allocation).

### Recruitment

All participants will be recruited in the three hospitals mentioned above through hospital-based advertisements. Once they have volunteered to participate, they will be assessed according to the inclusion and exclusion criteria. Eligible patients will be asked to sign the written informed consent form before intervention begins.

### Randomization, allocation, and blinding

The central randomization method will be performed by the Shanghai University of Traditional Chinese Medicine. Block randomization will be used, and the block size will be unknown to the researchers. The information of patients will be sent by an independent researcher to the center via the hospital’s website. Randomization will be performed automatically under the control of a central computer system. The researcher will be able to get the random numbers and group allocation immediately in the form of an email or short message service. Patients will be randomly divided into four groups with the ratio of 1:1:1:1.

This is a patient-blinded trial, based on treatment allocation. Physicians who prescribe the study medication and acupuncture therapy will not be involved in the outcome assessments or data analyses. All the outcome assessors and statisticians are blinded to the group assignment. Patients will wear an eye-mask during the acupuncture treatment, and each acupuncture treatment will be carried out in an enclosed space to ensure the successful implementation of the blinding method. Researchers will accept the training of the specifications of this study and be asked to adhere strictly to the principle of task separation.

### Intervention

During the trial, the participants are permitted to continue their regular medications including lipid-lowering drugs, antihypertensive drugs and drugs not related to the treatment of cognitive impairment. Drugs that may interfere with the efficacy of the study therapies will be banned such as cholinesterase inhibitor, non-steroidal anti-inflammatory drug (NSAID), etc.

Participants in the four groups will receive different treatments of acupuncture and herbal formula. Both the acupuncture and sham acupuncture treatments will be given twice a week for 8 weeks and then once a week for 4 weeks. Each treatment will last 30 min. A total of nine acupoints will be treated in the two therapies, including *Baihui* (DU20), *Shenting* (GV24), *Yintang* (GV29), *Sishencong* (EX-HN1), bilateral *Anmian* (EX-HN22), bilateral *Shenmen* (HT7), bilateral *Sanyinjiao* (SP6), bilateral *Hegu* (LI4), and bilateral *Taichong* (LR3). Participants will wear an eye-mask and lie in the supine position during the treatment. Needling will be performed after a routine skin disinfection. All the acupuncturists are licensed and have 3–5 years of experience in acupuncture treatment. Electrical stimulation will not be used in this trial.

The herbal treatment, YSG, will be given granules daily for 12 weeks, which is composed of 8 weeks of standard-dose followed by 4 weeks of mid-dose. The Chinese herbal formula, YSG, is composed of ten herbs, the main ones are *Rhizoma cyperi* 15 g, *Rhizoma acori tatarinowii* 9 g, *Radix polygalae* 9 g, as well as *Cortex cinnamomi* 3 g. The herbs will be mixed, cooked, filtered, and pressure-spray-dried to form the granules. The mid-dose YSG will contain half of the drug ingredient compared with the standard-dose YSG. All the granules will be packaged in single-dose sachets. Participants will receive individual packaged doses, with each dose to be dissolved in warm water and taken orally twice per day.

Both treatments will be performed by the same researcher. The granules will be provided every 4 weeks in a box; twenty-eight doses per box, three times in total. Participants will receive the study granule after the acupuncture treatment.

To ensure the adherence, the participants will be required to return the box before the new herbal medicine is provided. Participants who are unable to attend the appointment should contact the researchers in advance to reschedule. After all the treatments and assessments are completed, patients in all four groups will receive 400 RMB (about US$58) as a financial subsidy. All patients will, respectively, receive 200 RMB at the end of intervention and another 200 RMB at the end of the follow-up.

#### Group A

Participants in group A will receive acupuncture with YSG treatment. YSG was produced by a medical corporation (Beijing Tcmages Pharmaceutical co., Ltd., China). The real tube-needling (Wuxi Jiajian Medical instruments Co., Ltd., China) method will be used in group A. After insertion of needles, a lifting-thrusting manipulation or rotating manipulation will be applied to achieve a *De-Qi* sensation (a needling sensation of soreness, numbness, heaviness and/or distention). The acupuncture method is shown in Table 1.

**Table 1:** Acupuncture method

	Acupuncture treatment	Sham acupuncture treatment
Acupoints	DU20, GV24, GV29, EX-HN1, EX-HN22 (bilateral), HT7 (bilateral), SP6 (bilateral), LI4 (bilateral), LR3 (bilateral)	DU20, GV24, GV29, EX-HN1, EX-HN22 (bilateral), HT7 (bilateral), SP6 (bilateral), LI4 (bilateral), LR3 (bilateral)
Depth of needle insertion	DU20, GV24, GV29, EX-HN1, EX-HN22, HT7: 0.5 body-inches SP6: 1–1.5 body-inches LR3, LI4: 0.5–1 body-inches	No needle insertion
Response sought	*De-Qi* sensation	Without *De-Qi* sensation
Needle stimulation	Manual stimulation: lifting-thrusting manipulation or rotating manipulation will be applied to achieve a *De-Qi* sensation	No needle stimulation
Needle-retention time	Thirty min	Thirty min
Needle type	Stainless steel (0.30 × 25 mm or 0.30 × 4 0 mm; Wuxi Jiajian Medical instruments Co., Ltd., China)	Blunt stainless steel (0.30 × 25 mm or 0.30 × 40 mm; Wuxi Jiajian Medical instruments Co., Ltd., China)
Number of treatment sessions	Twenty treatment sessions in total	Twenty treatment sessions in total
Frequency and duration of treatment sessions	Twice per week for 8 weeks and followed by once a week for 4 weeks	Twice per week for 8 weeks and followed by once a week for 4 weeks
Other components of treatment	YSG or placebo herbal medicine	YSG or placebo herbal medicine

#### Group B

Participants in group B will receive acupuncture with placebo herbal medicine treatment. The placebo herbal medicine will be produced by the same company as YSG. The placebo is made of starch, caramel, coloring, and bitter flavoring, and will be given for 12 weeks in total. The appearance and taste of the placebo will be almost identical to the YSG.

#### Group C

Participants in group C will receive sham acupuncture with YSG treatment. The placebo acupuncture needle we adopt will be able to simulate an acupuncture procedure without penetrating the skin [19]. The patients will feel a pricking sensation when the tip of the blunt needle touches to the skin [20]. Meanwhile, the needle will retract inside the handle and appear to be shortened [20].

#### Group D

Participants in group D will receive sham acupuncture with placebo herbal medicine treatment.

### Outcomes

#### Primary outcome

The Montreal Cognitive Assessment (MoCA) is a one-page, 30-point test administered in 10 min [21]. The MoCA is a brief cognitive screening tool with high sensitivity and specificity for detecting MCI [21]. The current version tests eight cognitive functions: visuospatial functions, executive functions, memory, attention, and concentration, language, calculations, conceptual thinking, and orientation [22]. The Chinese version of the MoCA test is available from the following official website: https://www.mocatest.org/wp-content/uploads/2015/tests-instructions/MoCA-Test-Chinese_Beijing.pdf. Total scores of MoCA ranges from 0 to 30. A score of 25 or below indicates the possibility of cognitive impairment [22].

#### Secondary outcomes

The secondary outcomes refer to the MMSE and ERP.

The Mini-Mental State Examination (MMSE) is one of the most commonly used screening instruments for estimating the severity of cognitive impairment [23]. The MMSE is an 11-question measure that comprises five areas of cognitive function: orientation, registration, attention and calculation, recall, and language [24]. The total score of the MMSE ranges from 0 to 30. For a respondent with a high-school education and above, a score lower than 24 indicates possible cognitive impairment, and for a respondent with only a primary school education or who is illiterate, the cutoff scores are respectively 20 and 17 [25]. A lower score indicates a higher degree of cognitive impairment.

The event-related potential (ERP) is a kind of electrophysiological method with high temporal resolution [26]. ERPs have been used to examine the neurophysiological underpinnings of many conditions including MCI and AD [27]. P300 is one of the ERP components, which have been used to map the neural responses of memory [26]. Several subsequent memory effects, which measured activity differences that predict whether stimuli are later remembered or forgotten, are commonly observed as differences in P300 [28].

#### Adverse events

Adverse events (symptoms or diseases occurring during the trial) will be recorded and assessed at each session of intervention. The adverse events mainly include abnormal gastrointestinal reactions, allergic reactions, dizziness, itching after acupuncture, and other medical conditions. The relevance and severity of the adverse events will be assessed. Whether the participant could continue the treatment or not will be decided according to the assessments.

### Quality control

To ensure the quality of the trial, it will be conducted at three hospitals, and the trial data will be input on the ResMan website in a timely manner. The Clinical Research Center of Drugs of the Shanghai Municipal Hospital of Traditional Chinese Medicine will work as the data monitoring team to identify the existing problems in the project, control the bias, and make the final decision to terminate the trial. A qualified clinical trial expert will be invited to monitor this RCT. Researchers will be trained to ensure the quality of the cognitive tests. Acupuncturists participating in this trial are all certificated physicians with 3–5 years of clinical experience. To ensure that the acupuncture and sham acupuncture treatment are performed uniformly across the three hospitals, we will carry out centralized trainings twice for these acupuncturists before the trial begins.

### Statistical analysis

An intention-to-treat (ITT) analysis will be used in this RCT. Data analyses will be performed with the use of the statistical software SPSS20.0. The baseline characteristics of the participants in the four groups will be dealt with using the chi-squared test and Fisher Exact Test for categorical data and analysis of variance for continuous variables. Intragroup comparisons will be performed via the use of paired *t* test. The main effect and the interaction effect of the two interventions will be analyzed by 2 × 2 factorial analysis of variance. The statistical significance is set at *P* < .05. All the *P* values are two-side.

### Clinical trial registration

This trial is registered with Chinese Clinical Trials Registry (registration number: ChiCTR-INR-17011569, registered on 5 June 2017), and has been approved by the Ethics Committee of Shanghai Municipal Hospital of Traditional Chinese Medicine (2017SHL-KY-05).

## Discussion

It was reported that about 16% of adults diagnosed with MCI could revert back to normal or near-normal cognition approximately 1 year later [29]. However, those who reverted remain at higher risk for future cognitive decline than the individuals without a history of MCI [29]. Subjects with MCI also experience a greater mortality rate, compared with cognitively normal subjects [30]. The further development of MCI will affect the daily life of patients.

As a feasible complementary and alternative therapy, acupuncture has low side effects. We consider the regulation of the Governor Vessel to be the main acupuncture therapeutic principle of MCI. The two acupoints we chose (GV20, GV29) are two main acupoints of the Governor Vessel, which have proven to improve neurological functions. It was reported that stimulation at GV20 could alleviate the cognitive deficit and exert neuroprotective effects via modulation of the expression and processing of brain-derived neurotrophic factor in mice [31].

CHMs have long been used in China to treat cognitive decline. The YSG was developed according to the theory of “HuoXueShuGan” (invigorating the circulation of blood and smoothing the liver). *Rhizoma acori tatarinowii* and *Radix polygalae* in YSG are frequently used CHMs to treat memory impairment according to the traditional Chinese literature [32]. The extract of *Rhizoma acori tatarinowii*, which can be orally administrated, can promote the proliferation of neural progenitor cells [33]. Beta-asarone, the major active constituent of *Rhizoma acori tatarinowii*, has been shown to reverse the increase of apoptosis in the hippocampus of rats [34]. *Radix polygalae* extract, BT-11, has been reported to have memory-enhancing effects in healthy adults and in elderly humans [35, 36]. A recent study reported that polygalasaponin XXXII (PGS32), one of the active constituents of *Polygalae radix*, could attenuate scopolamine-induced cognitive impairments in mice [37].

A number of studies have shown the therapeutic effect of acupuncture and CHMs, respectively, on MCI. However, RCTs with good design are still lacking, and there has not been a clinical study on the combination of the two treatments for MCI yet. Accordingly, this trial has been designed as a multi-sited RCT to evaluate the therapeutic effects of acupuncture and YSG for elderly adults with MCI. Placebo and sham acupuncture will be used to evaluate the specific effects of YSG and acupuncture rather than the placebo effect for treating MCI. The appearance and taste of the placebo granules are almost identical to the YSG, and do not contain active ingredients. Since the therapeutic effect of superficial needling cannot be excluded [38, 39], non-penetration needling will be adopted as the sham acupuncture in the trial. Several evaluation tests will be used in this trial to optimize the accuracy of the evaluation. ITT analysis will be used to reduce the bias of this RCT. The Standards for Reporting Interventions in Clinical Trials of Acupuncture (STRICTA) [40] will be followed to ensure the quality control of the acupuncture treatment.

The design of this RCT still has limitations. MCI is considered to be a kind of chronic disease, thus a total treatment of 12 weeks may not be sufficient. We will develop a more reasonable treatment cycle and follow-up period according to the results of this trial. Besides, the acupuncturist could not be blinded to the acupuncture prescription of patients and its possible effects. Therefore, the acupuncturists will be asked to avoid discussing the treatment options with patients during the entire treatment. Furthermore, we chose a standard prescription instead of personalized therapy, which may lead to performance bias. This is because the study is designed to focus on the optimal acupuncture and CHMs treatments for MCI.

This RCT should provide us with information on the effect of treating MCI patients with only acupuncture, herbal formula or as a combination of both. The additive effect or synergistic effect of acupuncture and Chinese herbal formula will then be analyzed. The findings of this RCT are expected to provide evidence for evaluating the role of Chinese traditional treatment in the treatment of MCI.

## Trial status

We will start recruiting participants in June 2018.

## Additional files

Additional file 1: The SPIRIT checklist. (DOC 103 kb)

## Abbreviations

AD: Alzheimer’s disease
AMCI: Amnestic mild cognitive impairment
CDR: Clinical Dementia Rating Scale
CHMs: Chinese herbal medicines
DRS: Dementia Rating Scale
ERP: Event-related potential
GDS: Global Deterioration Scale
HAMD: Hamilton Depression Scale
ITT: intention-to-treat
MCI: Mild cognitive impairment
MMSE: Mini-Mental State Examination
MoCA: Montreal Cognitive Assessment
NSAID: Non-steroidal anti-inflammatory drug
RCT: Randomized controlled trial
STRICTA: Standards for Reporting Interventions in Clinical Trials of Acupuncture
YSG: *Yishen* Granule
